# Supplementary figures and images for: A citizen science approach to evaluating US cities for biotic homogenization
Source: PeerJ. 2019 Apr 30;7:e6879. doi: 10.7717/peerj.6879 (PMC6499060; doi:10.7717/peerj.6879)

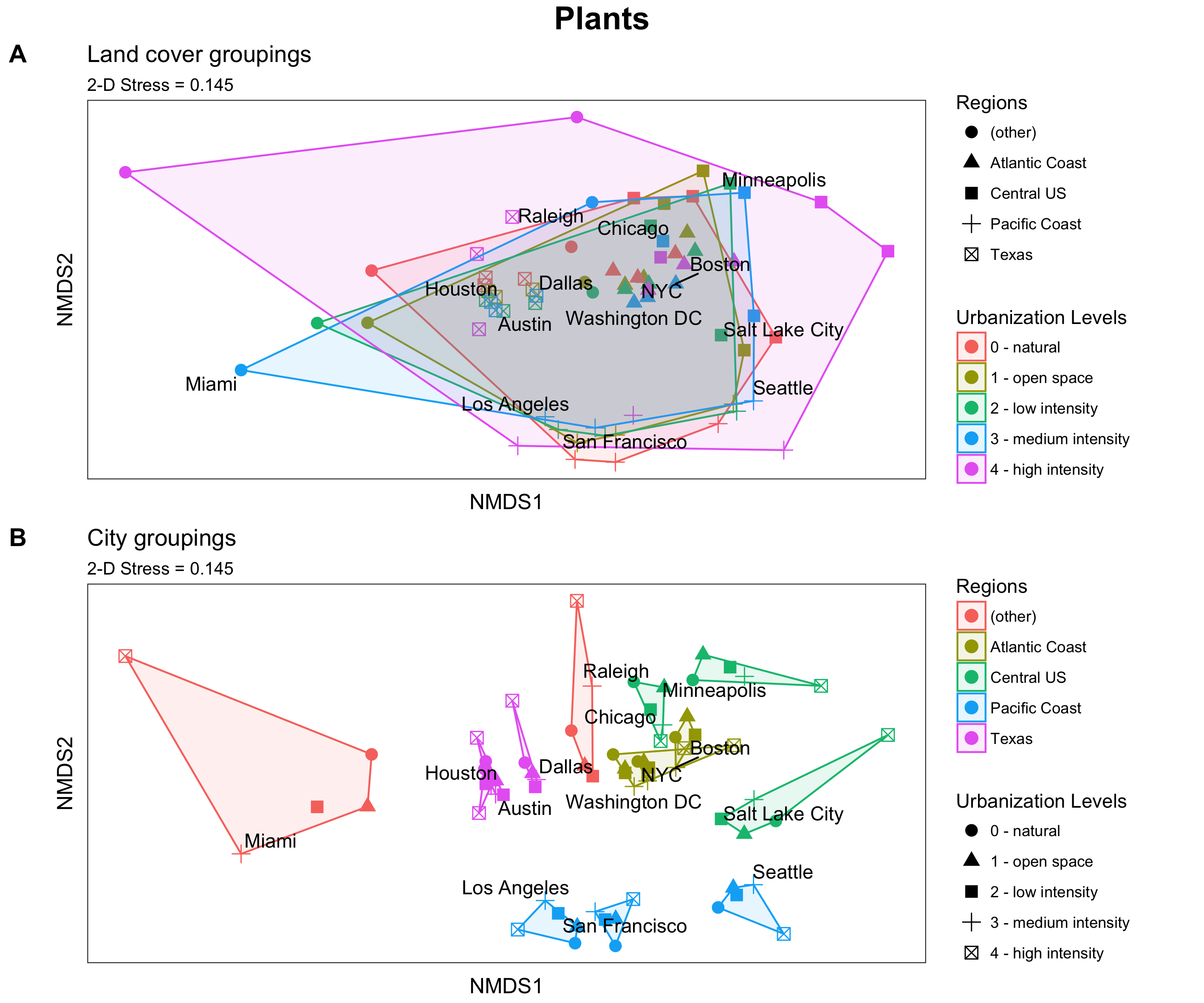

Supplement: Supplemental Information 1 — Built from a Bray–Curtis dissimilarity matrix, each point represents the community composition of a unique combination of one of the five urbanization intensity levels in one of the 14 cities. NMDS 2-D stress = 0.145. The two plots below are the same except different grouping visualizations are emphasized: in (A) points are grouped together by land cover type; in (B) points are grouped together based on city. [file peerj-07-6879-s001.png]
